# Cerebral Sinus Venous Thrombosis in a Patient Using Etonogestrel/Ethinyl Estradiol Vaginal Ring

**DOI:** 10.7759/cureus.3103

**Published:** 2018-08-05

**Authors:** Mohammad Selim, Amandeep Rakhra, Thamer Kassim, Rouhin Sen, Javaneh Jabbari, Carrie Valenta

**Affiliations:** 1 Internal Medicine, Creighton University School of Medicine, Omaha, USA; 2 Internal Medicine, Creighton University, Omaha, USA; 3 Internal Medicine, Creighton University Medical Center, Omaha, USA; 4 Internal Medicine, CHI Creighton University Medical Center, Omaha, USA; 5 General Surgery, Kansas University Medical Center, Kansas City, USA

**Keywords:** intracranial hemorrhage vaginal ring, contraceptive dural sinus thrombosis, dural sinus thrombosis, cerebral sinus vein thrombosis, intracranial hemorrhage vaginal ring, stroke, contraceptive dural sinus thrombosis, nuva ring stroke, nuva ring hemorrhage, dural sinus thrombosis, anticoagulation intracranial hemorrhage

## Abstract

A case of 43-year-old female presented to the emergency department (ED) with a new onset grand mal tonic-clonic seizure lasting at least two minutes with post-ictal confusion. Imaging was consistent with cerebral sinus venous thrombosis (CSVT) associated with intracranial hemorrhage. After ruling out most common causes of thrombosis, the etiology was attributed to estrogen vaginal ring. The patient was treated with anticoagulation therapy and had increasing hemorrhagic changes in the first few days, which eventually improved. The estimated annual incidence of cerebral sinus vein thromboses is approximately 3-4 cases per 1 million adults, mostly occurring in females. More than 80% of patients have favorable neurologic outcomes due to increased awareness of the condition as well as availability of advanced imagining and treatment options. The treatment is still controversial due to the high risk of intracranial hemorrhage with sinus thrombosis, especially for patients on anticoagulation. Still, most guidelines support starting anticoagulation. In this report, we highlight the association of CSVT with estrogen vaginal ring and discuss recent management recommendations per different society guidelines.

## Introduction

Presentations of cerebral sinus venous thrombosis (CSVT) can be diverse and range from a simple, non-specific headache to more complicated neurologic signs and symptoms. However, there is no specific clinical presentation that distinguishes CSVT from other neurological disorders. Therefore, diagnosis is quite difficult and requires a high clinical suspicion, especially in patients with underlying risk factors. In this case, we present a young adult female patient with idiopathic thrombosis of the right transverse dural venous sinus. No alternate underlying cause was identified; however, she was on etonogestrel/ethinyl estradiol vaginal ring which may increase the risk for CSVT.

## Case presentation

A 43-year-old female patient known to have a past medical history of depression, anxiety, and who used an etonogestrel/ethinyl estradiol vaginal ring for contraception, presented to the emergency department (ED) with new onset witnessed grand mal tonic-clonic seizure lasting at least two minutes with post-ictal confusion. The patient denied any previous seizure history. She did report drinking alcohol occasionally and had ingested two alcoholic drinks the previous evening. There was no associated trauma. Her vital signs on admission were: temperature 36.2ºC, pulse 119 beats per minute, respiratory rate 25 breaths per minute, blood pressure 140/105 mmHg, SpO2 99% on room air, and body mass index of 33.7 kg/m^2^. A thorough review of systems was negative other than nausea, diarrhea, and seizure. Physical examination, including a full neurological examination, was unremarkable. Laboratory data included: potassium 3.2 meq/L, bicarbonate 15 mmol/L, glucose 171 mg/dL, hemoglobin 11.5 g/dL, hematocrit 33.7%, platelet 134,000/mm^3^.

**Figure 1:**
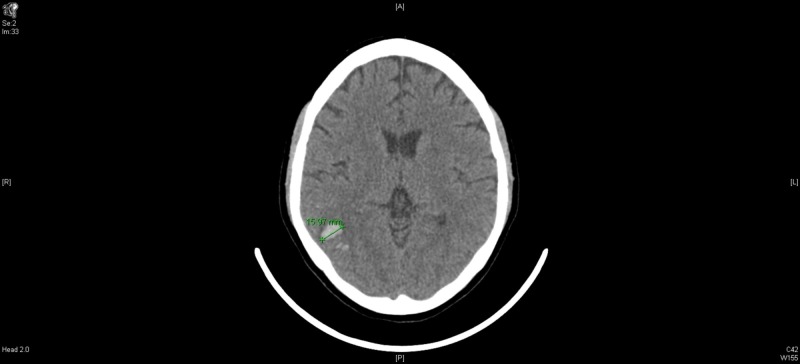
Computed tomography (CT) head without contrast, day 1.

Computed tomography (CT) head without intravenous contrast showed trace right parieto-occipital extra-axial collection and parenchymal hemorrhage of the right parieto-occipital and temporal regions, with an adjacent subarachnoid hemorrhage (SAH) (Figure 1). The acute right parieto-occipital intraparenchymal hemorrhage with scattered adjacent SAH was secondary to an extensive acute thrombosis of the right venous sinuses (transverse, sigmoid, and jugular). The patient was admitted to the intensive care unit (ICU) for further evaluation of her brain hemorrhage. She was started on nicardipine drip for a target systolic blood pressure of <140 mmHg and kept on hemorrhagic stroke protocol.

**Figure 2:**
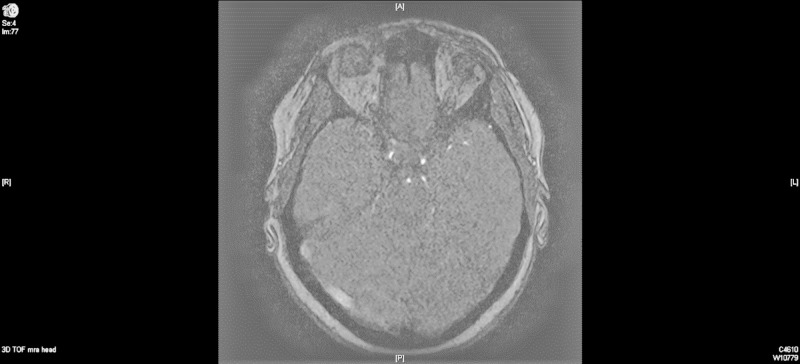
Magnetic resonance imaging (MRI) angiogram brain without contrast, day 1.

**Figure 3:**
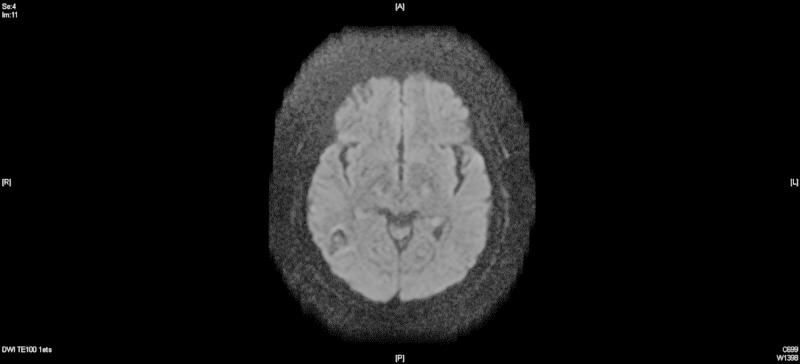
Magnetic resonance imaging (MRI) brain with contrast, day 1.

Neurology was consulted, and further evaluation included magnetic resonance angiogram (MRA) of the head without contrast. Findings were consistent with right transverse dural venous sinus thrombosis (Figure 2). Brain magnetic resonance imaging (MRI) with and without contrast showed intraparenchymal hemorrhage of the right parieto-occipital and temporal regions secondary to venous thrombosis. Patchy meningeal enhancement is seen at the right parieto-occipital and temporal regions, likely related to venous stasis versus reactive changes secondary to thrombosis. Also, a small focus of hyperintense signal is seen on isotropic diffusion-weighted and fluid-attenuated inversion recovery (FLAIR) sequences in the left splenium of the corpus callosum without significant associated decreased signal on apparent diffusion coefficient (ADC) maps, this is likely due to subacute ischemia (Figure 3).

**Figure 4:**
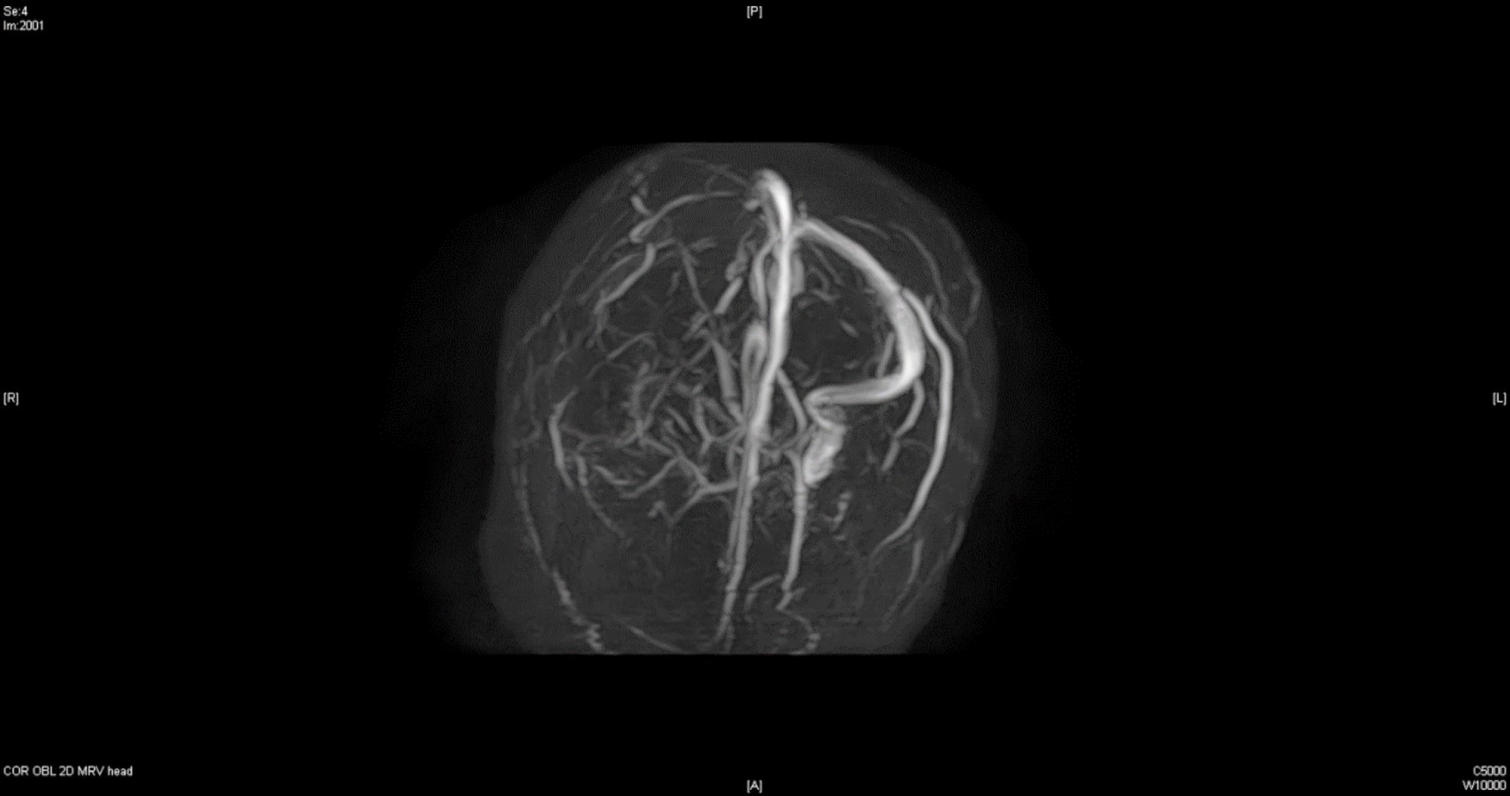
Magnetic resonance imaging (MRI) venogram brain without contrast, day 1.

Additionally, MRI venogram of the head without contrast showed a complete absence of flow-related enhancement in the right transverse and sigmoid sinuses and right internal jugular vein consistent with high-grade thrombosis of the right transverse sinus (Figure 4). Neurosurgery service was also consulted and recommended no surgical intervention.

**Figure 5:**
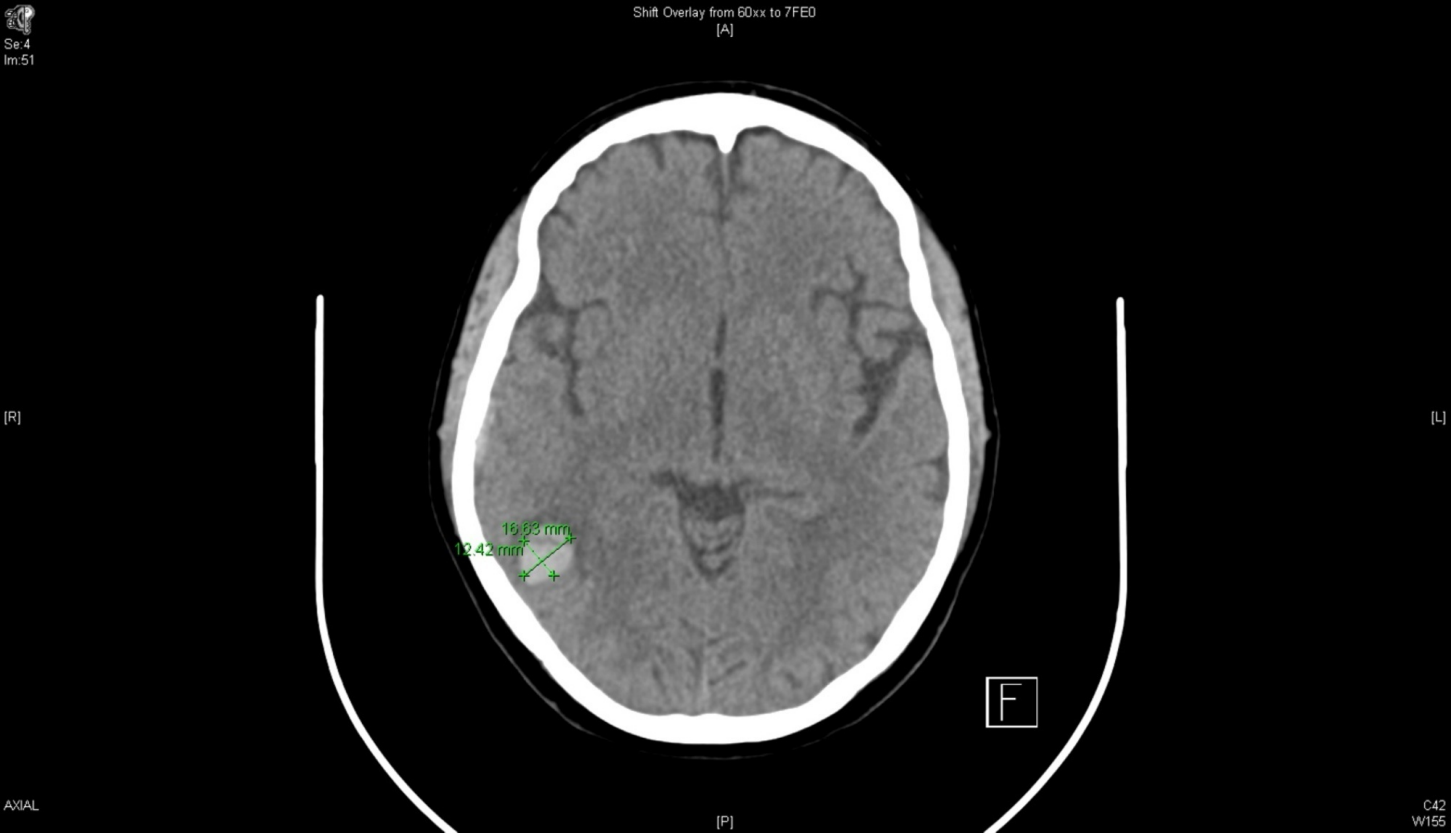
Computed tomography (CT) head without contrast, day 3.

**Figure 6:**
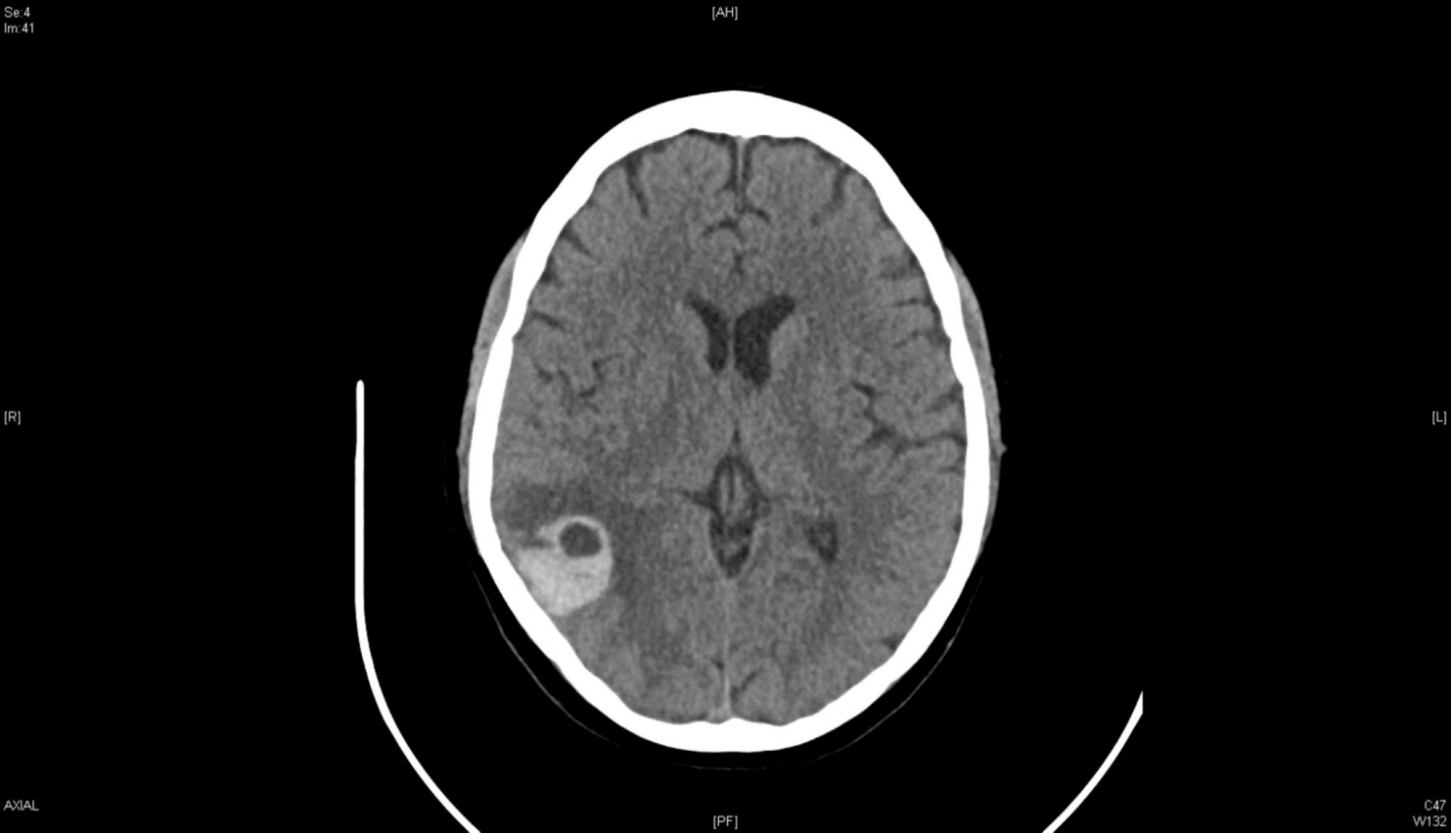
Computed tomography (CT) head without contrast, day 6.

On the third day of admission, after starting heparin drip, the patient complained of right ear fullness and increasing headache. A repeat head CT scan without IV contrast showed enlargement of the intracranial hemorrhage (Figure 5). Heparin infusion was held, with neurological assessments completed every hour. The patient was reassessed by neurosurgery and neurology teams, both of which recommended resumption of anticoagulation and starting warfarin for bridging with target international normalized ratio (INR) of 2-3. Once stable, the patient was transferred to a neurological center for further close follow-up and evaluation. The patient improved clinically over the following days and the cerebral hemorrhage improved on repeat CT imaging (Figure 6).

**Figure 7:**
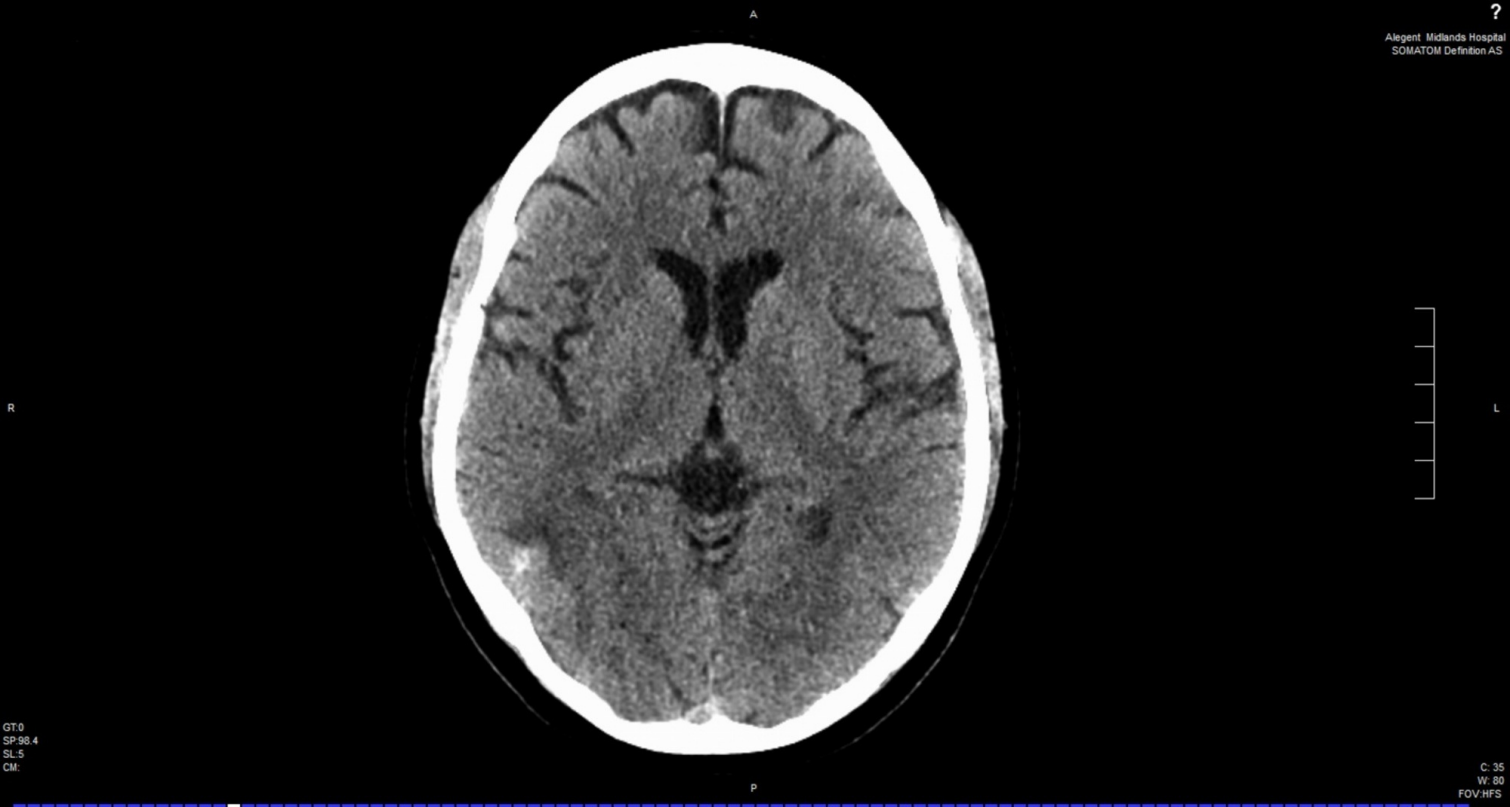
Computed tomography (CT) head without contrast six months later.

The patient was discharged home on warfarin with a plan to continue for a minimum of six months. On follow-up, six months later, the patient reported complete resolution of symptoms and CT head without contrast revealed significant improvement in the size of intracranial hemorrhage (Figure 7).

## Discussion

Thrombosis of the cerebral venous sinus is more common in those less than 50 years of age, with 75% of cases being found in women [1]. There is a wide range of CSVT etiologies that have been recognized which can be divided into two major categories: direct causes and hypercoagulable states. Direct causes, such as infection, were reported more frequently in the past. However, recent multi-center studies approximate incidence of direct causes at 8% to 12% of all cases annually [2-3]. Hypercoagulable states include factor V Leiden mutation, the presence of anticardiolipin antibody, antithrombin gene mutation, polycythemia, and hormonal contraceptives [4-5]. Peripartum and postpartum states are also considered hypercoagulable states and may contribute to the development of CSVTs, occurring in 12 out of 100,000 deliveries. A recent study published in March 2017 on Danish women aged 15-49 showed that etonogestrel/ethinyl estradiol vaginal ring (NuvaRing) carries a 2.5-time increased relative risk of thrombotic stroke when compared to nonusers [6].

The clinical presentation of CSVT is highly variable and most commonly non-specific. CSVT can present with diverse symptoms and signs that range from headaches of variable severity to seizures and hemispheric neurological manifestations. Rare cases have presented as brain stem herniation secondary to increased intracranial tension. Headache is the most frequent presentation but the least specific [4]. On the other hand, neurological manifestations are the most specific but less frequent signs [1]. Headaches typically increase gradually over days in most cases but sometimes over minutes to hours, mimicking the “thunderclap headache” of a potential subarachnoid hemorrhage. A rare presentation is the occurrence of unilateral hemispheric symptoms within days followed by symptoms of the opposite hemisphere.

The change in neurological deficits is likely caused by the development of a superior sagittal sinus cortical lesion on both sides of hemispheres. Up to 40% of patients with CSVT develop seizures; however, in most cases, they are limited, focal, and generally occur at the time of presentation or early in the disease course. Patients who present with seizure have a high risk of recurrent seizures within two weeks after the first seizure. Thalamic lesions may also occur in the setting of thrombosis of the deep venous system and typically present bilaterally. Lastly, behavioral symptoms such as delirium, amnesia, and mutism can also be presenting symptoms [4-5].

Diagnosis of CSVT must be based on high clinical suspicion, especially in middle-aged-female patients who present with unusual symptoms including new onset headache, stroke-like picture without obvious vascular risk factors, or patients with CT evidence of multiple hemorrhagic infarctions. The initial imaging test of choice is CT head to exclude other causes of the clinical presentation. There are direct and indirect types of characteristic findings seen on CT imaging. Direct signs include intravenous high-density shadow, “Band sign” (20-30% of cases) and “Empty δ sign” (16-46% of cases). Indirect signs include hemorrhagic cerebral infarction and brain edema. A negative CT scan, however, cannot exclude the diagnosis of CSVT. Magnetic resonance venography (MRV) is a highly sensitive test and can be used either alone or with MRI. The combination of both MRV and MRI would provide a CSVT diagnosis sensitivity reaching 90% or more [7]. CT venography (CTV) is a new modality that is promising in the diagnosis of CSVT. In the case of negative MRV, MRV/MRI, and CTV with high clinical suspicion, angiography would be the next best modality, especially in rare cases of cerebral venous thrombosis without sinus thrombosis [4,8].

The treatment goal is to recanalize the occluded sinuses, prevent thrombi from further propagation, and mitigate underlying etiologies, such as hypercoagulable states. However, there is some controversy around initiating anticoagulation due to the presence of hemorrhagic infarction occurring in up to 40% of cases at the time of presentation. Additionally, the remainder of cases have a high tendency to develop hemorrhages while receiving anticoagulation. Studies and meta-analyses are in favor of anticoagulation therapy despite the relative increased risk of hemorrhagic stroke. The current standard of care according to 2017 European Stroke Organization guidelines, 2014 American Heart Association and American Stroke Association (AHA/ASA) guidelines, and 2012 American Academy of Chest Physicians (ACCP) guidelines is to begin anticoagulation even in the presence of associated intracranial hemorrhage. Anticoagulation selection should be heparin or low molecular weight heparin followed by bridging to warfarin [8-11]. Oral anticoagulation should be given for 3-6 months if the CSVT was found to be secondary to transient factors for the first episode. It can be extended to 6-12 months in idiopathic etiologies. In cases of thrombophilia, especially severe forms of CSVT, or recurrent episodes, lifetime therapy is recommended [11-12]. The hypothesis is that hemorrhage in CSVT is caused by venous outflow blockage leading to both rupture of venules and to hemorrhagic transformation of venous infarctions. Many studies have been conducted to appreciate the effect of anticoagulation on patients with intracranial hemorrhages. These studies showed that the use of heparin and oral anticoagulation is relatively safe with some studies showing no incidence of hemorrhage and with others showing an increased risk of hemorrhage without a significant influence on patients’ outcomes [13-14].

In our patient, we initially treated her with intravenous heparin infusion without a loading bolus. On the third day of admission, the patient reported increased headache and right ear fullness. Repeat CT of the head was performed, showing worsening cerebral hemorrhage. The heparin infusion was held for hours but soon restarted after recommendations from neurology and neurosurgery and subsequently bridged to warfarin.

## Conclusions

CSVT is a rare condition that occurs more commonly in females younger than 50 years of age and presents with different neurological manifestations. Diagnosis requires a high clinical suspicion and different imaging modalities with high sensitivity, such as MRV/MRI. The standard of care for treatment, according to several guidelines, is heparin-based anticoagulation, followed by oral anticoagulation. It is recommended that treatment is continued for 3-6 months in the first episode if secondary to transient causes. Treatment with anticoagulation is safe in most cases, even in the presence of an intracranial hemorrhage, which may hypothetically be secondary to venous outflow blockage.
